# Exploiting sequence labeling framework to extract document-level relations from biomedical texts

**DOI:** 10.1186/s12859-020-3457-2

**Published:** 2020-03-27

**Authors:** Zhiheng Li, Zhihao Yang, Yang Xiang, Ling Luo, Yuanyuan Sun, Hongfei Lin

**Affiliations:** 1 School of Computer Science and Technology, Dalian University of Technology, Dalian, 116024 China; 2 School of Biomedical Informatics, University of Texas Health Science Center at Houston, Houston, 77030 USA

**Keywords:** Relation extraction, Document-level relation, Sequence labeling

## Abstract

**Background:**

Both intra- and inter-sentential semantic relations in biomedical texts provide valuable information for biomedical research. However, most existing methods either focus on extracting intra-sentential relations and ignore inter-sentential ones or fail to extract inter-sentential relations accurately and regard the instances containing entity relations as being independent, which neglects the interactions between relations. We propose a novel sequence labeling-based biomedical relation extraction method named Bio-Seq. In the method, sequence labeling framework is extended by multiple specified feature extractors so as to facilitate the feature extractions at different levels, especially at the inter-sentential level. Besides, the sequence labeling framework enables Bio-Seq to take advantage of the interactions between relations, and thus, further improves the precision of document-level relation extraction.

**Results:**

Our proposed method obtained an F1-score of 63.5% on BioCreative V chemical disease relation corpus, and an F1-score of 54.4% on inter-sentential relations, which was 10.5% better than the document-level classification baseline. Also, our method achieved an F1-score of 85.1% on n2c2-ADE sub-dataset.

**Conclusion:**

Sequence labeling method can be successfully used to extract document-level relations, especially for boosting the performance on inter-sentential relation extraction. Our work can facilitate the research on document-level biomedical text mining.

## Background

Semantic relations in texts can be expressed either intra-sententially (within a sentence) or inter-sententially (cross sentence boundaries). Inter-sentential relations can account for a substantial proportion and convey important meanings, especially in the biomedical domain. For example, in the paragraph “*Five of 8 patients improved during* ***fusidic acid*** *treatment: 3 at two weeks and 2 after four weeks. There were no serious clinical side effects, but dose reduction was required in two patients because of* ***nausea****.*”, the inter-sentential relation “*fusidic acid*”*-* induced “*nausea*” can be obtained only by integrating the semantic information in both sentences. However, most of the existing relation extraction methods [1–3] focus merely on intra-sentential relations, which is apparently insufficient in capturing inter-sentential ones.

**Fig. 1:**
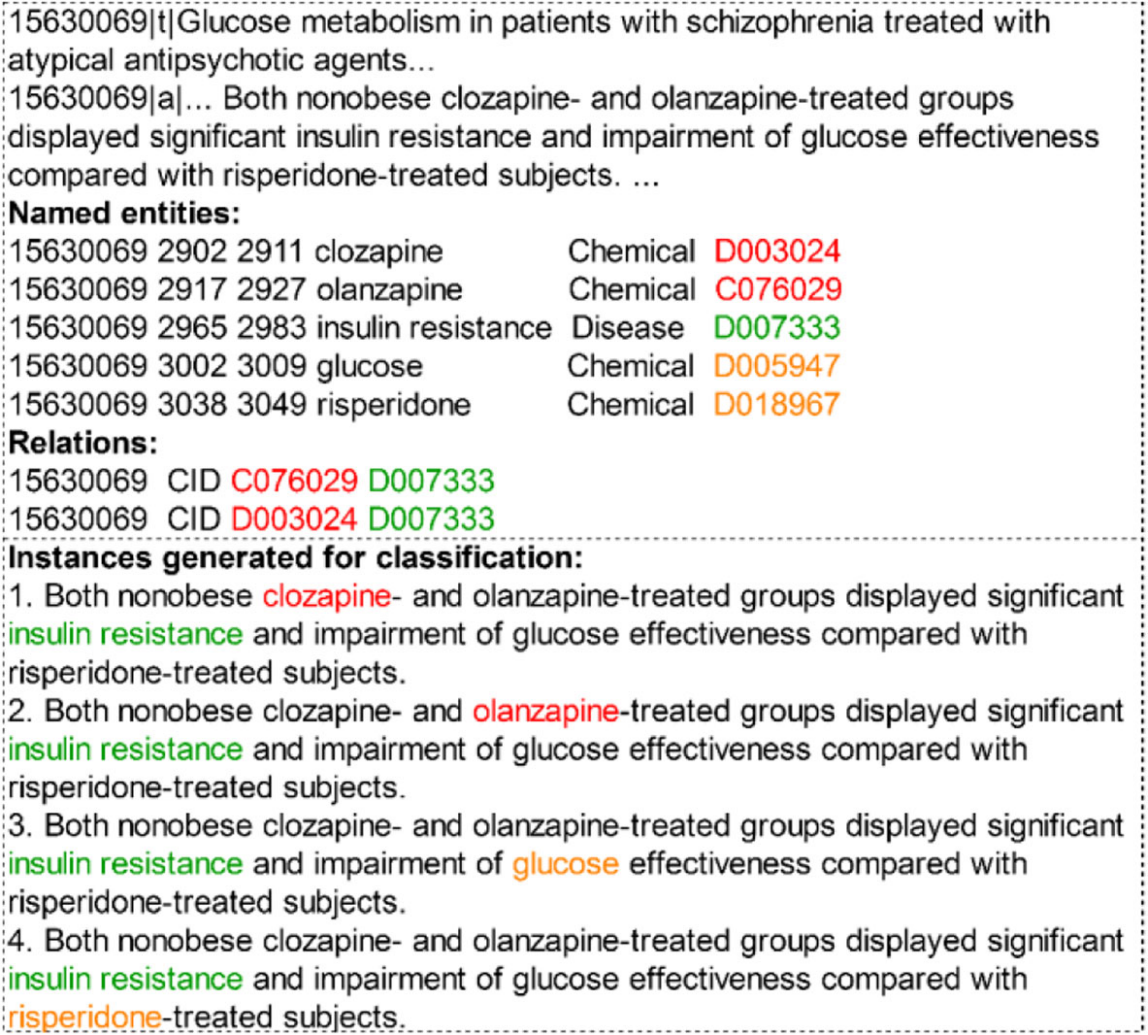
An annotated abstract in CDR corpus and instances constructed in classification-based methods. In the annotations, only related pairs are listed without specifying the exact entity offsets. There are 4 independent instances (entity pairs) constructed from the same sentence in the abstract according to classification-based methods

A benchmark document-level relation extraction task was proposed in the BioCreative V challenge, in which participating systems were asked to return all possible chemical-disease (CD) pairs that express document-level chemical-induced disease (CID) relations in a given abstract [4]. The upper portion of Fig. 1 shows an example of an abstract from the challenge corpus with its annotations. In this chemical disease relation (CDR) corpus, different from traditional sentence-level relation classification tasks (e.g. Semeval-2010 Task 8 [5]), the CID relations are annotated only at the document level (i.e. without giving the specific sentence that conveys a relation). For example, in Fig. 1, given an abstract, entity mentions with entity offsets and related entity pairs are annotated. According to the document-level annotation, it is hard to tell which sentence(s) convey(s) the meaning of a specific relation, since an entity can be mentioned multiple times in different sentences in an abstract and the offsets of related entities, which can be used to identify the unique mention of an entity in an abstract, are not given. In addition, the inter-sentential relations account for approximately 1/3 of all relations, signifying that traditional sentence-level relation extraction methods may not be appropriate to get satisfactory results.

In recent years, more and more researchers are aware of the importance of the document-level relation extraction. Preliminary works [6–8] adopt two classifiers to separately extract intra- and inter-sentential relations. In these methods, for the inter-sentential relations, inter-sentential instances are constructed from text spans that contain an inter-sentential entity pair and inter-sentential features are designed manually to classify the relation between the pair. Due to the complexity of multiple sentence expressions, it is much difficult to design inter-sentential features and thus, the inter-sentential classifier usually cannot achieve satisfactory performances. Besides, since these methods regard document-level relation extraction as a classification problem, instances even constructed from one sentence are independent. Therefore, during the training process, such classification-based methods only consider one entity pair at a time and neglect the relation interactions which are often helpful in determining relevant relations. For example, in Fig. 1 both chemicals “*clozapine*” and “*olanzapine*” have relations with “*insulin resistance*”. And the parallel relationship between “*clozapine*” and “*olanzapine*” can help recognize both relations and is called interactions between relations. In classification-based methods, the candidate relations “*clozapine*”-induced “*insulin resistance*” and “*olanzapine*”-induced “*insulin resistance*” are in two independent instances. Therefore, these classification-based methods cannot take the coordinate relation between “*clozapine*” and “*olanzapine*” into consideration.

More recent works [9–11] attempt to simultaneously extract intra- and inter-relations with only one classifier. They take multiple sentences (or an entire abstract) that contain the same entity pair as an input and output the document-level prediction. However, they are still unable to capture the interactions between relations. And the performance of inter-sentential relation extraction still needs to be improved.

To integrate the interactions between relations that are neglected by classification-based methods, we regard document-level relation extraction as a sequence labeling problem and propose a novel neural network method named Bio-Seq. Bio-Seq consists of a hybrid of feature extractors to generate document-level word representations and a conditional random field (CRF) layer to yield the final prediction for each word. The sequence labeling framework enables the identification of all the target entities related to a given source entity in a document, and thus integrates the interactions between relations. Consequently, according to the source and target entities, document-level relations are extracted.

The proposed method was evaluated on the CDR and the Adverse Drug Events (ADEs) Extraction in electronic health records (EHRs) of the 2018 National NLP Clinical Challenges (n2c2-ADE^1^) corpora, in which the relations are annotated at the document level and the mention level (i.e., each entity offset in a relation is annotated), respectively. Experimental results demonstrate that Bio-Seq achieves strong performances on both corpora.

The main contributions of our work are as follows:

1. We propose a sequence labeling-based method to integrate the interactions between relations for document-level relation extraction.
2. We design a hybrid of feature extractors to boost the performance on inter-sentential relation extraction.
3. We show that our method can achieve satisfactory generalization and outperforms other state-of-the-art methods at both document and mention levels.

## Related work

In the general domain, most of previous relation extraction works [12–14] regard relation extraction as a classification task and focus on extracting intra-sentential relations. These classification-based methods consider training instances as independent and neglect interactions between relations. By contrast, some works [15, 16] focus on integrating interactions between relations and thus employ sequence labeling methods to address relation extraction problems in which entity offsets are necessary to specify related entity pairs. Nonetheless, in some biomedical relation extraction tasks, the relations can be expressed intra- and inter-sententially and are annotated without giving entity offsets. Hence, the methods which take entity offsets as position features [13] in the general domain are not appropriate for biomedical document-level relation extraction.

In the biomedical domain, since entity offsets are unknown, early methods [7, 8, 17] generate intra- and inter-sentential instances according to the co-occurrence of an entity pair, i.e., any text span containing a co-occurring entity pair is labeled as positive if the entity pair is annotated, otherwise as negative. They mainly focus on extracting intra-sentential relations and investigate machine learning approaches with heavy feature engineering, assessing large-dimensional features derived from both the text itself and other external sources. As for inter-sentential relations, Gu et al. [7] and Gu et al. [8] built inter-sentential classifiers applying maximum entropy (ME) models while Zhou et al. [17] designed post-processing rules to identify them.

Recent works are inclined to effectively extract intra- and inter-sentential relations, simultaneously. On one hand, several studies [9, 10] adopt multi-instance learning (MIL) which aggregates multiple instances (regardless of intra- or inter-instances) containing the same entity pair into a candidate (bag) [18, 19] and assigns a relation label to that candidate. On the other hand, Zheng et al. [11] took an entire abstract with the chemical and disease mention tags of a CD pair as input and labeled the abstract as positive if the CD pair is annotated as related, otherwise as negative. Subsequently, they classified the abstract to determine whether the entities marked in the abstract is related to each other. Although these methods are able to extract document-level relations, they still neglect the interactions between relations and the performance on inter-sentential-level relation extraction needs to be improved.

Inspired by the general domain works, we regard relation extraction as a sequence labeling problem, which can integrate relation interactions that are neglected by classification-based methods. Also, different from above methods in the biomedical domain, we design multiple feature extractors at different levels to boost the performance on inter-sentential relation extraction.

## Methods

### Task description

Each document in the CDR corpus consists of a title and an abstract. It has been manually annotated with chemical, disease mentions associated with their Medical Subject Headings concept identifiers (MeSH® IDs) [4] and their document-level relations (i.e. only related pairs are listed without specifying the exact entity offsets). The goal of this task is to extract all the related CD pairs for each document.

**Fig. 2:**
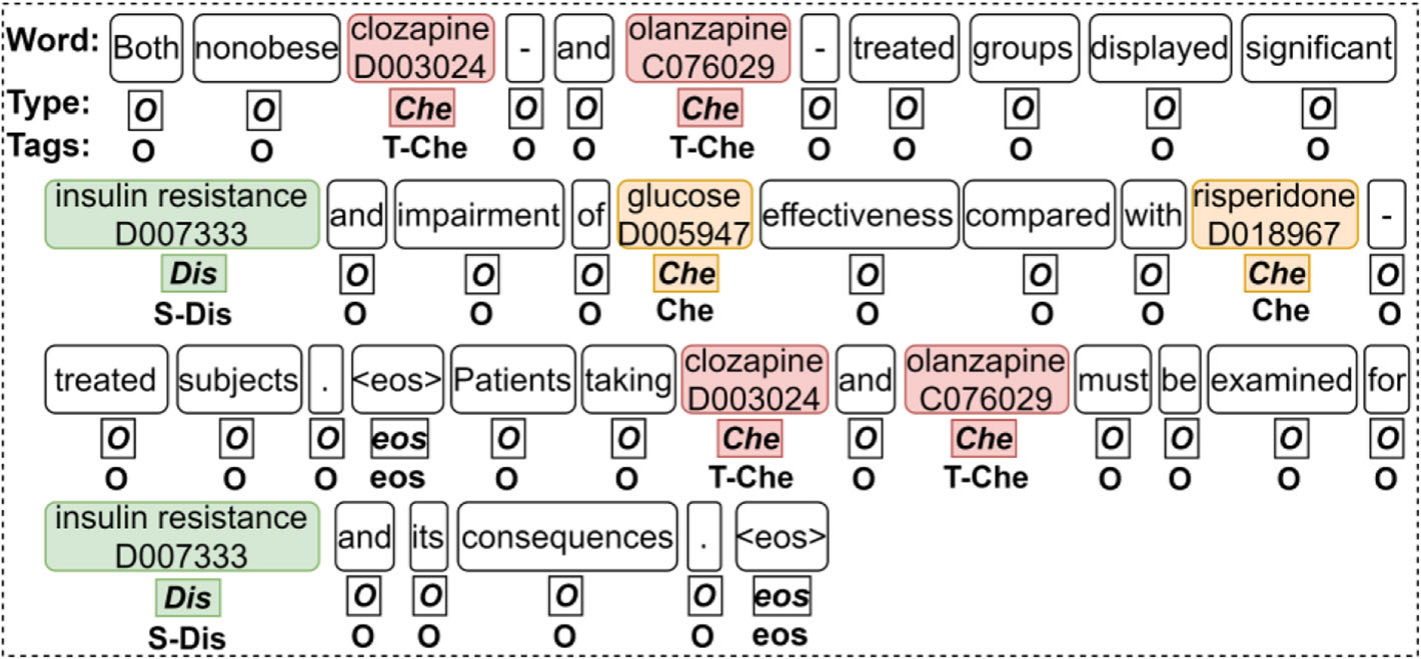
An example of input and output tags

In Bio-Seq method, given a document, each entity, whatever its type is, will be considered as a source entity. The model is trained to recognize all the target entities which have CID relations with the source entity and then construct relation pairs. In the method, the relation between a chemical and a disease will be confirmed as a positive relation only if a chemical is found related to a source disease while the disease is also found related to the chemical when it is regarded as a source entity in the corresponding instance. For example, Fig. 2 is an example when “insulin resistance” is regarded as a source disease and “clozapine” is labeled as a target chemical related to “insulin resistance”. And when “clozapine” is regarded as a source chemical and “insulin resistance” is labeled as a target disease by the method, the relation “clozapine-induced insulin resistance” is confirmed.

Specifically, the input of Bio-Seq is a combination of a word sequence and its corresponding type sequence. When generating a word sequence, all the entity mentions are replaced with their MeSH IDs, so as to normalize different mention expressions of an entity and keep its semantic meaning consistent across the expressions in the embedding layer. Besides, the “<eos>” tag is added at the end of each sentence to highlight sentence boundaries. A type sequence consists of the types of each word. We define six type categories including “Che”, “Dis”, “S-Che”, “S-Dis”, “<eos>” and “O” which denote “regular chemical”, “regular disease”, “source chemical”, “source disease”, “end of sentence” and “other”, respectively. The types are used to distinguish entities from regular words as well as a source entity from regular entities. For example, in Fig. 2 the type of “insulin resistance” is “S-Dis”, indicating that it is a source disease in the instance. Meanwhile, the chemical mentions “clozapine”, “olanzapine”, “glucose” and “risperidone” are labeled as “Che”.

The output tag set is defined as {“Che”, “Dis”, “S-Che”, “S-Dis”, “T-Che”, “T-Dis”, “O”, “<eos>”}. In the tag set, “Che” and “Dis” indicate that the word is recognized as a regular entity that has no relation with the source entity. “S-Che” and “S-Dis” indicate that the current word is recognized as the source entity in the input instance. “T-Che” and “T-Dis” indicate that the current word is labeled as the mention of a target entity. For example, in Fig. 2, “clozapine” and “olanzapine” are labeled as “T-Che” because they both have CID relations with the source disease “insulin resistance” according to document-level annotations. And other chemical mentions are labeled as “Che”, the words not in an entity are labeled as “O”, and the tag added at the end of each sentence is labeled as “<eos>”. Finally, the relation pairs between the source entity and each target entities are constructed. And since all the target entities are identified simultaneously, the interactions between relations, such as the coordinate relation between chemical “clozapine” and “olanzapine” in the sentence in Fig. 2 can be learned by Bio-Seq.

### Model overview

**Fig. 3:**
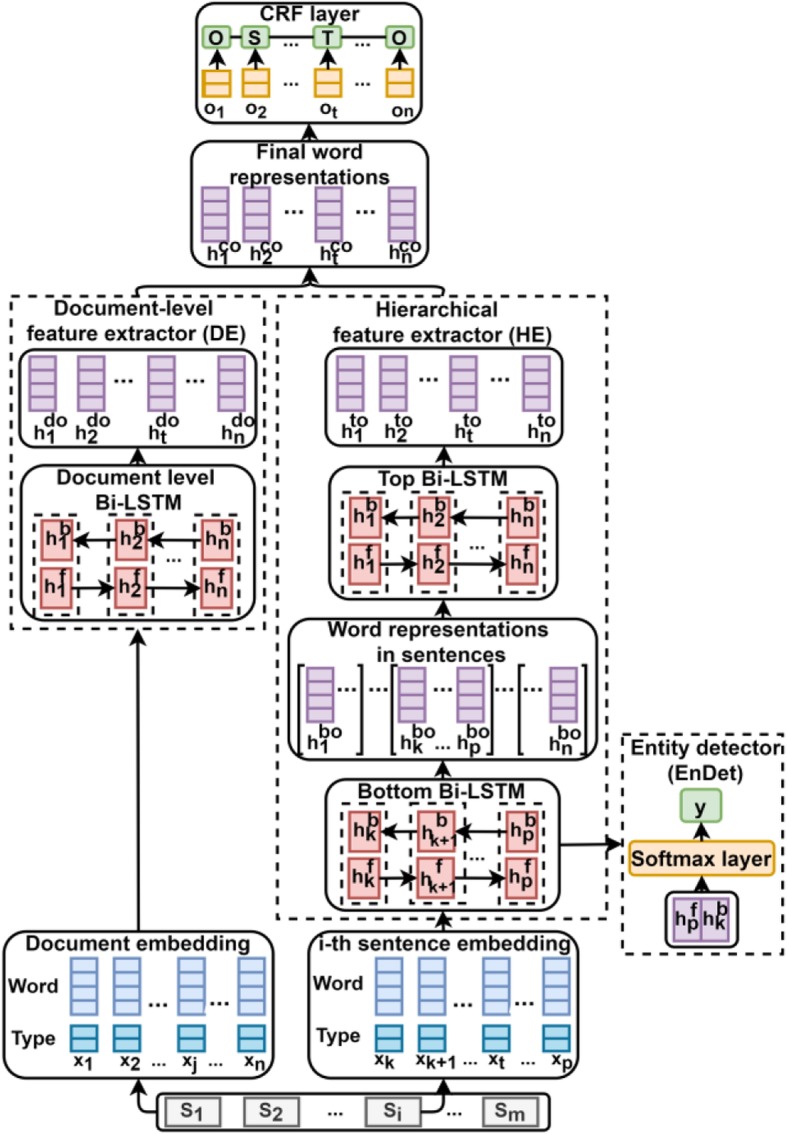
The architecture of the Bio-seq model

As shown in Fig. 3, Bio-Seq consists of two feature extractors: 1) a document-level feature extractor (DE) which generates word representations from an entire document and 2) a hierarchical feature extractor (HE) in which the bottom Bi-LSTM generates word representations at the sentence level while the top one subsequently concatenates all the word representations into a sequence and enables cross-sentence connections for the word representations. Inspired by previous classification methods in which entity position features [13] play a significant role in relation extraction tasks, we design an entity detector (EnDet) to emphasize the sentences that contain source and target entities. By sharing and training the parameters in the bottom Bi-LSTM network, entity location features are added to each word representation. Finally, the word representations generated by the two extractors are concatenated and fed into a CRF layer to yield the final predictions.

### Input representation

Given a document with the word sequence {w_1_, w_2_, …, w_n_} and type sequence {t_1_, t_2_, …, t_n_}, each word w_i_ and its type tag t_i_ are projected to corresponding embedding spaces, i.e., ${w}_i^{emb}$ and ${t}_i^{emb}$. Word embedding maps words into a low-dimensional space to capture semantic information among words [20] and it has been widely used to process the input of the neural networks in NLP tasks [13]. In this study, we employ the word2vec [21] tool to pre-train word embeddings using the texts that include chemical and disease annotations provided by PubTator [22] and the clinic notes in the Medical Information for Intensive Care (MIMIC)-III [23] for CDR and n2c2-ADE tasks, respectively. For the type feature, we map type tags to vectors and initialized them randomly. Thus, the overall embedding representation for word w_i_ is ${x}_i=\left[{\left({w}_i^{emb}\right)}^{\mathrm{T}},{\left({t}_i^{emb}\right)}^{\mathrm{T}}\right]$.

### Document-level feature extractor

In document-level relation extraction, the input sequence is a combination of a title and an abstract, which contains multiple sentences. Document-level feature extractor needs to process long sequences and captures essential features within a sentence and across sentences. Therefore, Bio-Seq uses a document-level Bi-LSTM network to generate word representations from a given document.

Long short-term memory (LSTM) is a variant of recurrent neural networks (RNNs) which can process sequential texts efficiently. It is designed by incorporating a separate memory cell with gating mechanism [24] to alleviate the gradient vanishing problem suffered by traditional RNNs when processing long sequences. For each element in the input sequence, the LSTM unit performs the following computations:

1 $$ {i}_t=\sigma \left({W}_{ii}{x}_t+{b}_{ii}+{W}_{hi}{h}_{\left(t-1\right)}+{b}_{hi}\right) $$

2 $$ {f}_t=\sigma \left({W}_{if}{x}_t+{b}_{if}+{W}_{hf}{h}_{\left(t-1\right)}+{b}_{hf}\right) $$

3 $$ {g}_t=\tanh \left({W}_{ig}{x}_t+{b}_{ig}+{W}_{hg}{h}_{\left(t-1\right)}+{b}_{hg}\right) $$

4 $$ {o}_t=\sigma \left({W}_{io}{x}_t+{b}_{io}+{W}_{ho}{h}_{\left(t-1\right)}+{b}_{ho}\right) $$

5 $$ {c}_t={f}_t{c}_{\left(t-1\right)}+{i}_t{g}_t $$

6 $$ {h}_t={o}_t\tanh \left({c}_t\right) $$

where *c*_*t*_ is the cell state at time t, and *i*_*t*_, *f*_*t*_, *g*_*t*_, *o*_*t*_ are the input, forget, cell, and output gates, respectively. σ is the sigmoid function.

In this study, we need to access both past and future input features for a given time, given the assumption that both forward and backward memories are informative. Thus, we utilize Bi-LSTM to generate word representations. Given a document {*x*_1_, *x*_2_, …, *x*_*i*_, …, *x*_*n*_}, the current word representation is a concatenation of the forward context representation ${h}_i^f$ which is computed by {*x*_1_, *x*_2_, …, *x*_*i*_} from left to right and the backward one ${h}_i^b$ which is computed by {*x*_*i*_, *x*_*i* + 1_, …, *x*_*n*_} from right to left. Finally, the document-level word representation is represented as ${h}_i^{do}=\left[{h}_i^f;{h}_i^b\right]$.

### Hierarchical feature extractor with an entity detector

Document-level feature extractor takes an entire document as input and may somewhat weaken the extraction of sentence-level features which are essential for identifying intra-sentential target entities. Therefore, to emphasize intra-sentential semantic features, Bio-Seq employs HE which consists of two Bi-LSTMs.

The input of the bottom Bi-LSTM is a sentence. First, we split a document into multiple sentences ending with the tag “<eos>” and then feed them into the bottom Bi-LSTM one by one. For example, {[*x*_1_, …, *x*_*i*_], [*x*_*i* + 1_, …, *x*_*n*_]} denotes a document with two sentences, and the output of the bottom Bi-LSTM is $\left\{\left[{h}_1^{bo},\dots, {h}_i^{bo}\right],\left[{h}_{i+1}^{bo},\dots, {h}_n^{bo}\right]\right\}$. Then we concatenate the sentence-level word representations into a sequence $\left\{{h}_1^{bo},\dots, {h}_i^{bo},{h}_{i+1}^{bo},\dots, {h}_n^{bo}\right\}$ and feed it into the top Bi-LSTM to enable cross-sentence connections for each word representation. Finally, the top Bi-LSTM outputs the hierarchical word representations.

As shown in Fig. 3, EnDet takes the sentence representation captured by the bottom Bi-LSTM to locate the source and target entity mentions. Given a sentence {*x*_1_, …, *x*_*i*_}, its representation is the concatenation of the last hidden state of both the forward and backward directions, i.e., $s=\left[{h}_i^f;{h}_1^b\right]$. Then it is fed into a fully-connected layer with a Softmax function to classify the sentence. The probability of a sentence belonging to a class is calculated as follows:

7 $$ \mathrm{p}\left(\mathrm{i}|s\right)= softmax\left({W}_o\bullet s+{b}_o\right) $$

where *W*_*o*_ and *b*_*o*_ are weight parameters, and *s* is the feature representation of a sentence. We define three classes to identify if source and target entity mentions exist in a sentence: 1) neither source nor target entity mentions exist; 2) only the source entity mention exists; 3) both source and at least one target entity mention exist.

### CRF layer

In Bio-Seq, the outputs of DE and HE are concatenated and then fed into a fully-connected layer to make independent tagging decisions for each word. Due to the fact that the tag of a word might also be affected by neighboring tags, instead of modeling tagging decisions independently, we use CRF model [25] to make use of neighboring tag information in prediction and decode the best tag path from all possible tag paths. In decoding, Viterbi algorithm [25] is used to get the predicted tag sequence.

During the training process, we first train the bottom Bi-LSTM and EnDet with sentences. Cross-entropy loss function is applied to calculate the gradient and update the parameters. Then we train the two feature extractors and the CRF layer to assign a tag to each word.

## Results and discussions

### Datasets and experimental settings

The Bio-Seq method is evaluated on two datasets: the CDR and n2c2-ADE corpora, which model relations between chemicals and diseases at the document level in biomedical literature and between drugs and ADEs at the mention level in clinical notes, respectively. Table 1 lists the statistics of the two corpora. For the n2c2 corpus, we only took the paragraphs that contain ADEs and regarded other drug mentions (e.g. “twice a day” which is annotated as a frequency entity) as regular words. In n2c2-ADE, approximately 1/6 of all relations are inter-sentential. We randomly split 20% of the original training set of n2c2-ADE corpus into a development set.

**Table 1:** The statistics of the CDR and n2c2-ADE corpora

Dataset	Documents	Relations	Intra-sentential relations	Inter-sentential relations
CDR				
Training	500	1038	755	283
Development	500	1012	766	246
Test	500	1066	763	303
Total	1500	3116	2284	832
n2c2-ADE				
Training	243	873	695	178
Development	60	219	167	52
Test	202	733	607	126
Total	505	1825	1469	356

Precision (P), recall (R) and F1-score (F1) are used to evaluate the performance of our method. The F1-score is defined as F1 = 2∙P∙R/(P + R), which can quantify the overall performance by balancing precision and recall.

We used the Pytorch library [26] to implement our proposed method. The dimensionalities of word embedding and type embeddings are set as 100 and 30, respectively. The hyper-parameters are tuned on development sets and finally set as follows: the number of hidden units of the document-level, bottom and top Bi-LSTM is 150, 100 and 150, respectively, and the mini-batch size is set as 32. To alleviate overfitting, we used dropout [27] to randomly drop units and their connections and the dropout rates of the embedding layer and the bottom Bi-LSTM output layer are set as 0.2 and 0.5, respectively. In the training process, adaptive moment estimation (Adam) [28] is used to optimize the objective function parameters and the learning rate of Adam is set as 0.001. All the results of our method are averaged over 10 runs with 10 random seeds.

### Comparisons with the state-of-the-art methods

We compare our method with both two-level and document-level classifiers on the CDR corpus in Table 2. CD-REST [6] utilizes two classifiers to extract document-level relations and obtains the best performance in the BioCreative V challenge in 2015. Besides, Gu et al. [7] and Gu et al. [8] applied an inter-sentential classifier, while Zhou et al. [17] exploited post-processing (pp) rules to identify inter-sentential relations. All the methods above focus on feature engineering. By contrast, RPCNN [9], BRAN [10] and Zheng et al. [11] are document-level NN-based classifiers which automatically extract both intra- and inter-sentential relations without using handcrafted features and generally obtain better performance than two-level classifiers.

**Table 2:** Comparison between our method and other state-of-the-art methods on CDR corpus

System	Method	Concept level	P	R	F1
CD-REST [6]	SVM + SVM	Sen + Doc	0.596	0.440	0.507
Gu et al. (2016) [7]	ME + ME	Sen + Doc	0.620	0.551	0.583
Zhou et al. [17]	LSTM-SVM	Sen	0.649	0.493	0.560
	LSTM-SVM + pp	Sen + Doc	0.556	0.684	0.613
Gu et al. (2017) [8]	CNN + ME	Sen + Doc	0.609	0.595	0.602
	CNN + ME + pp	Sen + Doc	0.557	0.681	0.613
RPCNN [9]	CNN-RNN	Doc	0.552	0.636	0.591
BRAN [10]	Transformer	Doc	0.499	0.638	0.555
	Transformer-NER	Doc	0.556	0.708	0.621
Zheng et al. [11]	LSTM-CNN	Doc	0.543	0.659	0.595
	LSTM-CNN + pp	Doc	0.562	0.680	0.615
Bio-Seq	LSTM-CRF	Doc	0.600	0.675	**0.635**

It is shown in Table 2 that our Bio-Seq method achieves the best F1-score (0.635) without applying any feature engineering or post-processing rules. There is a 12.8% improvement compared with that of CD-REST (0.507). Also, there is a 1.4% improvement compared with BRAN which applies multi-task learning to boost the performance and conducts the best F1-score (0.621) on the CDR corpus. Besides, compared with other document-level classifiers, Bio-Seq achieves the highest precision and there is an almost 5% improvement. These observations verify the effectiveness of Bio-Seq.

Table 3 lists the results on n2c2-ADE corpus. Different from the CDR corpus, the n2c2 corpus is annotated at the mention level with specified entity offsets. Since the results of the n2c2 challenge have not been made public available, the baseline model (CNN-LSTM^2^ in Table 3) used for comparison is built based on a popular NN-based framework and the results are averaged by 5-fold cross-validation. The model exploits a hybrid of a CNN and a Bi-LSTM layers to generate a sentence representation which is subsequently fed to a fully-connected layer with a Softmax function to classify the relation. Since it is a sentence-level model which may miss the inter-sentential relations, two post-processing rules are designed to recall inter-sentential relations: 1) if an ADE is not labeled as relevant to any drugs, it will match the nearest drug to construct a drug-ADE relation; 2) if the ADE and the drug in a predicted relation exist in different sections (e.g. the drug exists in MEDICATIONS and the ADE exists in DIAGNOSIS), the relation will be removed.

**Table 3:** Results on n2c2-ADE corpus

Model	P	R	F1
CNN-LSTM	0.814	0.700	0.753
CNN-LSTM + pp	0.813	0.809	0.811
Bio-Seq	0.892	0.814	**0.851**

As shown in Table 3, the post-processing rules demonstrate their effectiveness through increasing the recall by 10.9% while maintaining the precision. In comparison, our Bio-Seq method achieves better performance (especially with a much higher precision (0.892 vs. 0.813)) without using any rules. Bio-Seq learns the relation interactions between multiple target entities (regardless of where the entity exists in the document) simultaneously, which helps to ensure the effectiveness and accuracy of the features, allowing the model to recall more accurate relations, especially inter-sentential relations.

Overall, the results show that our method is competitive or superior in performance, compared with other state-of-the-art methods used for document-level relation extraction from both biomedical literature and clinical notes.

### Results at the intra- and inter-sentential levels

Table 4 lists the results at the intra- and inter-sentential levels. A CD pair would be labeled as positive if the relation between the pair is annotated at the document level, otherwise as negative. CD pairs that are not involved in any intra-sentential instances are considered as inter-sentential ones.

**Table 4:** Results of intra- and inter-sentential relations on CDR corpus

System	Intra-sentential results			Inter-sentential results		
	P	R	F1	P	R	F1
Gu et al. (2016) [7]	0.674	0.689	**0.682**	0.514	0.298	0.377
Gu et al. (2017) [8]	0.597	0.550	0.572	0.519	0.070	0.117
Zheng et al. [11]	0.595	0.779	0.674	0.450	0.429	0.439
Bio-Seq	0.634	0.720	0.674	0.518	0.573	**0.544**

We observe that Gu et al. [7] achieves the highest F1-score at the intra-sentential level owning to large-dimensional feature engineering, but a lower one at the inter-sentential level. In contrast, our Bio-Seq method is much more balanced and achieves the best F1-score (0.544) at the inter-sentential level with a comparable one at the intra-sentential level (0.674). Compared with Gu et al. [7] and Gu et al. [8], it achieves 16.7 and 42.7% improvements of F1-score at the inter-sentential level, respectively. The reason is that inter-sentential relations are expressed by spanning multiple sentences, and discourse inferences such as coreference resolution might be needed when extracting such relations. Therefore, it is difficult to design inter-sentential-level features and feature-based models usually may achieve worse performances. In contrast, NN-based methods learn features from data using a general-purpose learning procedure [29] so that they can capture more complex features and achieve satisfying generalization.

In addition, our Bio-Seq method possesses significant advantages over Zheng et al. [11] at the inter-sentential level with an 10.5% improvement of F1-score. Also, its precision at the intra-sentential level is 3.9% higher than that of Zheng et al. [11]. The possible reasons are as follows: 1) Bio-Seq exploits a sequence labeling-based framework which takes all the target entities into consideration and can simultaneously encode multiple entities pairs, while Zheng et al. [11] only considers one pair of entities at a time and neglects the interactions between relations. 2) Bio-Seq aims to distinguish target entities from regular ones, rather than to capture the features of expressing a CID relation such as whether the verb “induce” exists in the context or not. Thus, the features are more specific and effective to recognize inter-sentential target entities than those captured by classification-based methods. 3) The document sequence is too long for Zheng et al. [11] to generate one representation without losing essential features and the fixed width of hidden vectors becomes a bottleneck when the Bi-LSTM models must propagate dependencies over long texts [30]. Therefore, taking an entire document as an input and generating a fixed length representation for the document will not be appropriate for relation classification problem.

### Effectiveness analysis on each component

To further verify the effectiveness of each component of Bio-Seq on both corpora, we removed a component (or components) each time and then calculated the corresponding decrement on Bio-Seq’s F1-score.

Table 5 shows the results on the CDR corpus after removing components. It can be observed that DE plays an essential role in extracting both kinds of relations, especially for the inter-sentential ones. A significant decrease of F1-score (11.4%) at the inter-sentential level demonstrates the ability of DE in handling inter-sentential relation extraction. Although the top Bi-LSTM layer of HE enables cross-sentence connections, DE can capture inter-sentential features more directly. Also, since the input of the top Bi-LSTM is the representations captured by the bottom one, some intra-sentential information may already be filtered at the sentence level. Therefore, only applying HE is insufficient for document-level relation extraction. Moreover, when EnDet is removed, the recall at intra- and inter-sentential levels decreases by 0.9 and 2.4%, respectively. It verifies that EnDet is capable to recall both kinds of relations because it emphasizes the sentences which contain the source and target entities. In addition, when HE and EnDet are removed, the overall recall drops by 1.4% and the intra- and inter-sentential-level recalls drop by 0.8 and 3%, respectively, which also demonstrates the effectiveness of the combination of DE and HE.

**Table 5:** The overall and intra- and inter-sentential level results of different component evaluated on CDR corpus. ∆ denotes the corresponding F-score decrease percentage when a component is removed

Component(s) removed	Overall				Intra-sentential level				Inter-sentential level			
	P	R	F1	∆(%)	P	R	F1	∆(%)	P	R	F1	∆(%)
None	0.600	0.675	0.635	–	0.634	0.720	0.674	–	0.518	0.573	0.544	–
-DE	0.554	0.603	0.577	−5.8	0.592	0.683	0.634	−4.0	0.444	0.417	0.430	−11.4
- EnDet	0.599	0.647	0.622	−1.3	0.637	0.696	0.665	−0.9	0.507	0.533	0.520	−2.4
-DE & EnDet	0.551	0.611	0.579	−5.6	0.587	0.692	0.635	−3.9	0.446	0.420	0.433	−11.1
-HE & EnDet	0.609	0.633	0.621	−1.4	0.648	0.684	0.666	−0.8	0.513	0.515	0.514	−3.0

In conclusion, the sequence labeling framework is suitable for extracting document-level relations, and the multi-level feature extractors can emphasize valuable intra- and inter-sentential features which further boost the performance effectively.

## Conclusion

Existing classification-based methods for document-level relation extraction fail to effectively extract inter-sentential relations and neglect the interactions between relations in a document. To address these problems, we regarded document-level relation extraction as a sequence labeling task and proposed a novel method Bio-Seq to extract document-level relations directly. The results showed that Bio-Seq outperforms other state-of-the-art models on both biomedical literature and clinical notes. Compared with other NN-based models, Bio-Seq can learn more distinguishable features between related and regular entities, and thus, is capable of accurately extracting relations by integrating interactions between relations. In addition, the multiple feature extractors boosted the performance of extracting inter-sentential relations by recalling more positive ones.

## Data Availability

The corpus of BioCreative V chemical disease relation corpus can be downloaded at: https://biocreative.bioinformatics.udel.edu/tasks/biocreative-v/track-3-cdr/ The n2c2 challenge have not been made public available. Details can be found at: https://n2c2.dbmi.hms.harvard.edu/participate

## Abbreviations

CD: Chemical-disease
CID: Chemical-induced disease
CDR: Chemical disease relation
CRF: Conditional random field
ADE: Adverse drug event
n2c2: National NLP Clinical Challenges
ME: Maximum entropy
MIL: Multi-instance learning
MeSH® ID: Medical Subject Headings concept identifier
DE: Document-level feature extractor
HE: Hierarchical feature extractor
EnDet: Entity detector
MIMIC: Medical Information for Intensive Care
LSTM: Long short-term memory
RNN: Recurrent neural network

## Footnotes

1 https://n2c2.dbmi.hms.harvard.edu/participate

2 This method is shared by the winner of the n2c2–2018 relation extraction challenge
